# A facility-based “brain gym”: feasibility and preliminary effectiveness of a long-duration, low-frequency dual-task and exergaming intervention in older adults

**DOI:** 10.3389/fpsyg.2026.1767634

**Published:** 2026-03-26

**Authors:** Ryan M. Glatt, John A. Bettencourt, Dawn Kirk, Sue Paul, Karen J. Miller, Prabha Siddarth

**Affiliations:** 1 Pacific Neuroscience Institute and Foundation, Santa Monica, CA, United States; 2 Department of Statistics and Applied Probability, Department of Mathematics, University of California, Santa Barbara, Santa Barbara, CA, United States; 3 Asbury Methodist Village, Gaithersburg, MD, United States; 4 Department of Psychiatry and Biobehavioral Sciences, David Geffen School of Medicine, University of California, Los Angeles, Westwood, CA, United States

**Keywords:** cognition, cognitive-motor training, dual-task training, exercise, exergaming, older adults, rehabilitation, serious games

## Abstract

**Introduction:**

Exercise programs that combine cognitive and physical challenges may support cognition in aging populations. This single-arm feasibility study evaluated the implementation and acceptability of a 12-month, facility-based dual-task training (DTT) and exergame (EXG) exercise program in older adults residing in a senior living community.

**Methods:**

Secondary exploratory analyses examined preliminary effectiveness on cognitive outcomes. Seventy-five residents aged 65 years and older participated in a long-duration, low-frequency program combining DTT and EXG interventions in a senior living community, completing 8–24 sessions over a 12-month period. Cognitive outcomes were assessed using the Montreal Cognitive Assessment (MoCA), CNS Vital Signs (CNSVS), and a subjective memory questionnaire. Within-subject changes were examined using non-parametric tests and effect sizes.

**Results:**

Among the 75 participants enrolled in the program (mean age = 82.1 years; SD = 5.6, 61% female), feasibility endpoints were met, with 75% adherence to ≥16 sessions and no adverse events were reported. Exploratory analyses demonstrated statistically significant improvements in global cognition (MoCA; *p* < 0.0001, Cohen’s *d* = 0.61), composite cognitive performance (CNSVS; *p* < 0.0001, *d* = 0.45), and in selected measures of processing speed and executive functioning. No dose–response relationship was observed between the number of sessions attended and the change in cognitive outcomes.

**Discussion:**

These findings suggest that a long-duration, low-frequency dual-task and exergame-enhanced program can be feasible and safe in a senior living setting and demonstrates preliminary evidence of cognitive benefit. Findings should be interpreted cautiously, given the single-arm design and the absence of a control group.

## 1 Introduction

Non-pharmacological interventions for older adults, including those with cognitive impairment, neurological conditions, and healthy aging populations, have demonstrated potential to slow the progression of cognitive decline (Falck et al., 2019; Denkinger et al., 2012). Among lifestyle approaches, exercise is consistently recognized as one of the most beneficial and accessible strategies for reducing or mitigating the risk of dementia (Small, 2016), and its benefits extend to individuals with existing cognitive impairment (Law et al., 2020; Demurtas et al., 2020; Loprinzi et al., 2018). While exercise is associated with improvements in memory, executive functioning, and overall physical health, evidence remains inconclusive as to whether exercise alone is sufficient to address the diverse range of cognitive processes affected in aging (Snowden et al., 2011).

Recent research has shifted toward multicomponent interventions that integrate cognitive and motor challenges (Manser et al., 2023). A systematic review by Tait et al. (Karr et al., 2014) found that simultaneous training, where physical and cognitive tasks are combined in real time, was more effective for cognition than sequential training. Herold et al. (Tait et al., 2017) similarly argued that embedding cognitive demands into motor tasks may yield greater neurocognitive benefits than addressing them separately. Evidence supports modest yet significant improvements in cognitive-motor outcomes through concurrent training, with benefits observed in healthy older adults (Herold et al., 2018; Bamidis et al., 2014), individuals with mild cognitive impairment (MCI) and dementia (Zhu et al., 2016; Law et al., 2014; Karssemeijer et al., 2017), and populations with Parkinson’s disease or multiple sclerosis (Werner et al., 2018). This is particularly relevant, as dual-task ability, which involves performing two distinct tasks concurrently, declines with age and neurological disease, correlating with cognitive decline and an increased risk of falls (Fritz et al., 2015).

### 1.1 Dual-task training interventions

Dual-task training (DTT) involves the simultaneous execution of two independent cognitive and motor tasks, with one task typically designated as primary (e.g., walking) and the other as secondary (e.g., verbal fluency) (Plummer et al., 2015). Unlike complex single tasks, where multiple demands are nested within a single activity, true dual-task paradigms require the division of attentional and executive resources across distinct, goal-directed tasks (Plummer et al., 2015).

Evidence supports the use of DTT as an effective approach for improving both physical and cognitive outcomes across diverse populations. Fritz et al. (Werner et al., 2018) reported significant benefits for gait velocity, stride length, balance, and cognition in individuals with Parkinson’s disease and Alzheimer’s disease following dual-task training. Plummer et al. (Oh and Yang, 2010) further highlighted that physical exercise interventions targeting gait-related dual-task interference reduce fall risk among older adults.

DTT methods are diverse with examples including cognitive tasks while walking (verbal fluency, serial subtractions, or arithmetic processing) (Chao et al., 2014), memory recall during mobility (recalling word lists, names of people) (Wollesen et al., 2020), dynamic tasks (catching or throwing a ball while maintaining balance) (McCallum and Boletsis, 2013), progressive resistance with concurrent counting (Benzing and Schmidt, 2018), or conversational or autobiographical recall while moving (Stanmore et al., 2017). Other approaches extend into group fitness settings, such as Dual-Task Zumba Gold, where rhythmic dance movement is paired with counting or memory challenges (Stojan and Voelcker-Rehage, 2019), square-stepping programs requiring both coordination and memory for choreography (Jhaveri et al., 2023), or obstacle negotiation with auditory discrimination (Adcock et al., 2019).

Declines in dual-task ability, as measured by assessments such as the Timed-Up and Go Cognitive (TUG-C) condition, are predictive of declines in physical and cognitive function, including dementia conversion among individuals with MCI (Norouzi-Gheidari et al., 2013), gait variability in neurodegenerative conditions (Lauzé et al., 2017), and fall risk (Barry et al., 2016). Interventions that address dual-task performance may directly translate to improved independence and reduced morbidity. Existing evidence suggests that combined cognitive-motor training found in DTT is more effective in improving cognition than exercise alone (Bamidis et al., 2014; McIsaac et al., 2015).

### 1.2 Exergaming interventions

Exergaming (EXG) refers to interactive digital systems that require physical movement to play and provide real-time visual and/or auditory feedback, combining physical activity with game mechanics. Serious exergames, in contrast to recreational entertainment games, are intentionally designed for health, rehabilitation, or performance enhancement purposes. These systems may enhance engagement, provide adaptive feedback, and support graded task progression beyond traditional exercise formats (Yogev-Seligmann et al., 2012). Within the Games for Health taxonomy, EXGs can be categorized as general (e.g., Wii Sports, designed for broad physical and cognitive engagement without targeting specific outcomes (de Andrade et al., 2013)) or specific (e.g., games designed to train particular motor or cognitive domains (Schwenk et al., 2010)). Clinical EXGs are applied in therapeutic contexts, sometimes referred to as “Rehabilitainment,” which combines rehabilitation and entertainment (Coelho et al., 2013) through specific exergames. EXGs vary across multiple dimensions, including intensity, frequency, technology utilization, cognitive complexity, and social interactivity (Evans et al., 2009). These characteristics enable tailoring to specific populations, ranging from community-dwelling healthy older adults to those with neurological conditions, such as Parkinson’s Disease (Parial et al., 2021; Wang et al., 2021).

The current evidence base supports general and clinical EXG as both feasible and effective, with meta-analyses confirming that exergaming can improve executive functioning, attention, and visuospatial abilities in both healthy and clinical populations (Stanmore et al., 2017). Older participants often perceive EXGs as less physically strenuous than equivalent gym-based exercise, even when the physiological intensity is comparable (Montero-Odasso et al., 2017), providing a rationale for utilizing EXGs to increase physical activity intensity and adherence.

Clinical EXG devices with relevance to older adults with and without cognitive impairments include the SMARTFit system (SMARTFit Inc., Camarillo, CA), which delivers LED light-based stimuli (letters, numbers, characters) that must be identified (reacting, matching, locating) and struck with the upper-extremity or held implement while performing simultaneous physical actions (such as balancing or marching) (Pieruccini-Faria et al., 2021); the Dividat Senso (Dividat AG, Schindellegi, CH), which trains stepping strategies alongside executive function, memory, and processing speed through the engagement of reaction-based exergames (Åhman et al., 2020); and platforms such as LudoFit (Ludica Health, Montreal, QC, CA), which use motion-capture or webcam tracking to deliver exergame-based training for both upper and lower extremities in either clinical or home environments, demonstrating improvements in gait, motor control, and engagement (Lauenroth et al., 2016; Bond et al., 2021). Clinical EXG is a promising, sustainable, and engaging intervention that may further bridge the gap between physical activity and cognitive training.

### 1.3 Integrating exergaming and dual-task training

Although many EXGs include cognitive-motor elements, they do not always meet the definition of DTT, as responses may be sequential rather than concurrent (Plummer et al., 2015). For example, striking a light stimulus may engage reaction speed but not simultaneous cognitive demands. EXGs may be beneficial in isolation, but programs combining EXG and DTT may be more effective (Manser and de Bruin, 2021), although this has yet to be empirically demonstrated. The FitBrain program (Pacific Neuroscience Institute and Foundation, Santa Monica, CA) exemplifies this model, combining SMARTFit, Dividat Senso, and LudoFit technologies with DTT methods (Glatt et al., 2024), which may be more easily scaled to community-based settings (Chua et al., 2021), although this remains largely unexplored (Pieruccini-Faria et al., 2021; Rieker et al., 2022).

Cognitive gains from EXG and DTT interventions can persist for weeks or months after training, with stronger effects observed in adults over 60 and those with lower baseline cognition (Gouveia et al., 2020; Erickson et al., 2019). Sex may also moderate outcomes, with larger improvements reported in studies with women (Gui et al., 2024). Combined interventions generally yield greater benefits, especially for executive function, compared with single-modality training (Gouveia et al., 2020; Gates et al., 2020). While computerized cognitive training effects often wane without continued practice (Gavelin et al., 2021; Manser et al., 2024), DTT and EXG interventions show more durable improvements, particularly in executive function and memory, with some retention months after cessation (Gouveia et al., 2020). These findings highlight the potential of multimodal, cognitively engaging exercise programs to support sustained cognitive health in older adults.

### 1.4 Rationale and objectives

Although a growing body of research supports the cognitive and physical benefits of DTT and EXG as separate components (Tait et al., 2017; Fritz et al., 2015; Stanmore et al., 2017), most interventions have been brief, isolated, and conducted in limited settings (Jhaveri et al., 2023; Adcock et al., 2019). Few structured, long-term, facility-based multimodal programs that incorporate both DTT and EXG interventions have been developed and systematically evaluated, particularly within senior living communities (Lauzé et al., 2017; Glatt et al., 2024). This gap limits an understanding of how sustainable, multimodal programs can be delivered at scale in real-world contexts and whether such approaches confer measurable benefits to cognition in community-dwelling older adults.

The primary aim of this study was to evaluate the feasibility of implementing a long-duration, low-frequency DTT and EXG intervention in a senior living community. Feasibility endpoints included adherence, retention, and safety. Secondary exploratory aims examined preliminary effectiveness on global cognition, domain-specific cognitive performance, and subjective memory. Three secondary exploratory cognitive outcomes were selected, based on prior literature and studies (Tait et al., 2017; Herold et al., 2018; Bamidis et al., 2014; Zhu et al., 2016; Law et al., 2014; Karssemeijer et al., 2017; Werner et al., 2018; Fritz et al., 2015; Lauenroth et al., 2016): (a) global cognition, as measured by the Montreal Cognitive Assessment (MoCA); (b) domain-specific cognitive functions, including processing speed, executive functioning, and memory, as assessed by CNS Vital Signs (CNSVS) computerized cognitive assessment; and (c) subjective memory ratings, used to capture individuals’ perceptions of changes in their everyday cognitive functioning. It was hypothesized that participants would demonstrate improvements in objective cognitive measures; however, these analyses were exploratory.

## 2 Methods

### 2.1 Study design and setting

This study was conducted as a single-arm feasibility study with exploratory secondary effectiveness outcomes, consistent with the framework for pilot and feasibility studies described by Eldridge et al. (2016). Feasibility outcomes were defined *a priori* as adherence ≥70% of planned sessions, retention ≥80%, and absence of serious adverse events. Cognitive assessments were conducted at baseline and approximately 12 months after participation in a community-based program. The program took place at *Asbury Methodist Village* in Gaithersburg, Maryland. The intervention was structured as two separate 8-week programs, each comprising approximately eight sessions, delivered over 12 months.

Participants were offered 16–24 total supervised DTT & EXG training sessions (8-week segments, with a maximum of 24 total sessions over the course of a year), implemented in community activity and wellness spaces within the facility. Participants completed a long-duration, low-frequency program consisting of 8–24 total sessions delivered across two 8-week training blocks over a 12-month period. The facilitators of the intervention were NCCA-accredited Certified Personal Trainers with experience working with older adults and received additional training in DTT & EXG under the oversight of an Occupational Therapist. All sessions were delivered in person with continued supervision and a staff-to-participant ratio of 1:6. Attendance was recorded at each session.

### 2.2 Participants

Seventy-five older adults were recruited from the facility’s residents for the program. Eligibility criteria included age ≥65 years, residence at Asbury Methodist Village, ability to ambulate independently with or without an assistive device, and willingness to participate in scheduled group sessions. Exclusion criteria comprised uncontrolled cardiovascular or metabolic conditions, severe visual or auditory impairments preventing task participation, advanced dementia or neuropsychiatric illness, and inability to provide informed consent. Recruitment strategies included informational sessions, flyers, and staff referrals. This single-arm feasibility study involving human participants was reviewed and approved by The Institute for Evaluation and Research, LLC (“TIER”), located in Kansas City, MO. TIER determined the program met criteria for exemption because it was classified as a program evaluation of an existing wellness service, involved minimal risk, and used non-invasive, standard functional and cognitive assessments. The exemption was granted because the program constituted evaluation of an existing wellness service rather than a research intervention introducing additional risk. Participants provided verbal agreement to participate in the wellness program; written research consent was not required under the IRB exemption determination.

### 2.3 Intervention protocols

The intervention, which represents a combination of serious EXG and DTT interventions, integrated both traditional DTT and clinical EXG devices, such as the SMARTFit, Dividat Senso, and LudoFit. DTT tasks required participants to execute simultaneous cognitive and motor tasks, such as maintaining balance while performing serial subtraction, walking while naming words in a particular category, or throwing and catching a ball while alternating letters. Sessions lasted approximately 60 min and were delivered about once weekly during two separate 8-week training blocks across a 12-month period, representing a long-duration, low-frequency model. This intervention framework was adapted from the previously described “FitBrain program” (Glatt et al., 2024) and modified for delivery in a community-based senior living environment. The primary tools utilized included technologies such as SMARTFit (Jhaveri et al., 2023; Chua et al., 2021), the Dividat Senso (Manser et al., 2023), and LudoFit (Glatt et al., 2024), as well as non-technological DTT approaches, such as naming tasks during balance or gait tasks (Silsupadol et al., 2006).

Each session included a 5–8 min warm-up (low intensity, median RPE = 2) involving range-of-motion, marching, and simple orientation or recall tasks; approximately 40 min of circuit-style training (moderate intensity, median RPE = 4–6); and a 5–8 min cool-down (median RPE = 1–2) consisting of breathing and stretching exercises and low-demand cognitive engagement. During the circuit phase, participants rotated through 4–6 stations, each lasting about 5–10 min at a moderate intensity (median RPE = 4–6). Stations remained conceptually consistent across sessions but varied in cognitive complexity and physical intensity. Fidelity was maintained through standardized session templates. An example of the different stations and session structure is shown in Table 1.

**Table 1:** Sample session structure.

Exercise name	Duration	Sets	Physical movement	Cognitive domain focus	Description
Warm-up	8 min	1	Marching, range-of-motion	Basic attention	Practice range-of-motion tasks for major joints and muscle groups; marching; overview session structure
SMARTFit—track the target	5 min	2	Static balance	Sustained attention, visual processing speed	Hit target as fast as possible, maintain static balance (feet together)
Senso—simple	2.5 min	2	Dynamic balance	Sustained attention, visual processing speed	Step to red target as quickly as possible. Hold on to handrail as needed.
SMARTFit—seek the color	5 min	2	Dynamic balance	Visual processing speed, selective attention	Hit the correct color as fast as possible while performing a lateral step (left to right).
Senso—targets	2.5 min	2	Dynamic balance	Sustained attention, visual processing speed	Step on targets at right time, focusing on accuracy. Hold on to handrail as needed.
LudoFit—skiing	2.5 min	2	Dynamic balance	Sustained attention, visual processing speed	Focus on weight-shifting and collecting as many stars as possible.
Dual-Task—Serial 3’s	2.5 min	2	Obstacle walking	Attention and processing speed	Navigate obstacles while counting backwards by 3’s out loud from a random starting number (200+)
Cool-down	8 min	1	Stretching	Basic attention	Breathing, stretching exercises, session review

Progression and individualization were based on participant performance, safety, and perceived exertion. Initial task difficulties and movements were selected based on baseline individual capabilities and individualized progression was based on safety and performance. Cognitive load was increased by increasing the game level (which often increased speed or complexity), introducing task-switching or inhibitory rules, or through adaptive levelling (e.g., games increase in difficulty based on individual performance). Complexity in cognitive tasks was increased by emphasizing a different cognitive domain across tasks; earlier sessions focused on attention (e.g., extinguishing illuminated targets of a specific color) and processing speed tasks (e.g., hitting targets as fast as possible), whereas later sessions focused on executive functioning (e.g., sequencing targets in an order) and memory tasks (e.g., matching or recalling sequences).

Physical intensity was progressed through faster stepping, increased movement amplitude, or reduced hand support. Difficulty increased when participants demonstrated consistent task accuracy (≥80%) with safe movement execution and decreased when fatigue, balance concerns, or frustration were observed. The intervention incorporated behavior change principles, including graded task progression, real-time performance feedback, social facilitation through group delivery, instructor reinforcement, and perceived competence enhancement.

These elements align with constructs from Social Cognitive Theory (mastery experience and observational learning) and Self-Determination Theory (competence, autonomy, and relatedness), which may contribute to adherence and sustained engagement (Manser et al., 2023; Manser and de Bruin, 2021; Manser et al., 2024). No modifications to the intervention protocol were made during the study period. The intervention was reported in accordance with the Consensus on Exercise Reporting Template (CERT) (Slade et al., 2016). A completed checklist is provided in Supplementary material 3.

### 2.4 Outcome measures

Feasibility outcomes were predefined prior to program implementation, and included adherence, retention, and safety. Adherence was defined as the proportion of participants attending at least 16 sessions across the two 8-week training blocks. Retention was defined as the proportion of enrolled participants who completed follow-up cognitive assessments within 12 months of the initial training period. Safety was evaluated by monitoring and documenting adverse events during all training sessions. These feasibility endpoints were defined *a priori* based on thresholds commonly used in feasibility studies of exercise interventions (Slade et al., 2016) in older adults, including adherence ≥70%, retention ≥80%, and absence of serious adverse events (Lauzé et al., 2017; Chua et al., 2021; Eldridge et al., 2016).

Exploratory and secondary cognitive outcomes included the MoCA (Nasreddine et al., 2005) to assess global cognition, the CNSVS (Gualtieri and Johnson, 2006) fixed-order computerized battery to assess processing speed, executive function, visual memory, and verbal memory, and a subjective memory questionnaire. MoCA and CNSVS have established reliability and validity in older adult populations (Nasreddine et al., 2005; Gualtieri and Johnson, 2006). Subjective memory performance was measured using the computerized Memory Questionnaire (MEMQ) subjective memory questionnaire, provided within CNSVS, in which lower scores indicate better subjective memory performance across daily tasks (Supplementary material 1) (Gualtieri and Johnson, 2006; Royle and Lincoln, 2008).

Assessments were conducted by trained program facilitators within a dedicated senior living community room with minimal distractions proximal to the intervention space. The individuals who facilitated assessments were also the intervention program facilitators (qualified fitness staff supervised by an occupational therapist). The intervention took place in a small fitness room within the senior living community dedicated to DTT and EXG interventions. Assessments were conducted at baseline and repeated at the 12-month endpoint.

### 2.5 Statistical methods

The participants’ demographics were summarized using descriptive statistics. Demographic characteristics that were normally distributed are summarized using means and standard deviations. Violin plots paired with boxplots were used to visualize the distribution of the cognitive outcomes at baseline and post-intervention. Shapiro–Wilk tests and visual inspection of distribution plots indicated non-normal distributions for several outcome variables; therefore, these variables are presented using medians and interquartile ranges and analyzed using non-parametric methods. This approach ensures that summary statistics and inferential tests are aligned with the data’s distributional properties.

No formal power calculation was performed, as this was an exploratory feasibility study. Adherence was defined as attending ≥16 of 24 sessions. The secondary exploratory outcome measures were the MoCA, CNSVS Composite, and subjective memory. The CNSVS subscales were examined if the Composite score showed a statistically significant change (Oh and Yang, 2010). Within-person changes in these outcomes from baseline to post-intervention were examined using Wilcoxon signed-rank tests. For each outcome, we report the Hodges–Lehmann (HL) median paired difference with 95% CIs as the primary effect size (typical change in original units); to aid interpretability and for comparability with prior literature, we also report parametric effect sizes (Cohen’s *d*). These effect sizes were interpreted using conventional thresholds (0.2 = small, 0.5 = medium, 0.8 = large). To adjust for baseline and covariates, we modeled the 12-month score as a function of baseline score, age, sex, and program dose. Our primary specification was median (*τ* = 0.5) quantile regression with percentile bootstrap CIs, which is robust to skew and outliers.

We also examined whether changes in outcomes were associated with the number of sessions participants completed. Dose–response was evaluated in two ways. First, we treated dose as a 3-level factor. We used the Kruskal-Wallis test to determine whether there was a significant association between changes in cognitive outcomes, and the number of sessions completed by participants. Second, we treated dose as an ordinal variable (8, 16, 24 sessions) in the baseline-adjusted model to estimate a linear trend per 8-session step. Three secondary exploratory outcomes (MoCA, CNSVS Composite, and subjective memory) were selected and tested at a two-sided *α* = 0.05. CNSVS subscales were examined only if the Composite score was significant. All statistical analyses and data visualizations were performed using R (v4.4.2).

## 3 Results

### 3.1 Demographics

Seventy-five participants were enrolled and completed the low-frequency one-year program (median days = 371.5, range: 161–758, mean = 370.8, SD = 141.8). The predefined feasibility thresholds were met: 75% (56 of the 75) of participants attended at least 16 sessions, and no serious adverse events occurred during the intervention period. Participants ranged in age from 64 to 93 years old, with a mean age of 82.1 (median = 83, range: 64–93, SD = 5.6), and approximately 61% were women (Table 2). Education levels were generally high, with 34 (45%) participants having a bachelor’s degree, 37 (49%) having a master’s degree, and 4 (5%) having a doctoral degree. Participants completed between 8 and 24 sessions (mean = 14.6, SD = 4.4, median = 16). Detailed baseline characteristics are presented in Table 2.

**Table 2:** Descriptive statistics of participants.

Variables	Total
Number of participants	75
Sex, Female *n* (%)	46 (61.3)
Age, years, mean (SD)	82.1 (5.6)
Education level, *n* (%)	34 (45.3) Bachelor’s
	37 (49.3) Master’s
	4 (5.3) Doctorate
Race, *n* (%)	71 (94.7) White
	4 (5.3) Asian
Baseline MoCA, mean (SD)	26.9 (2.6)
Possible Dementia (MoCA < 19), *n* (%)	1 (1.3)
MCI (MoCA 19–24), *n* (%)	9 (12)
No MCI (MoCA > 24), *n* (%)	65 (86.7)
Number of sessions attended, mean (SD)	14.6 (4.4)
Number of participants who attended 8 sessions (%)	19 (25.3)
Number of participants who attended 16 sessions (%)	50 (66.7)
Number of participants who attended 24 sessions (%)	6 (8)

### 3.2 Feasibility

Predefined feasibility thresholds were met. Seventy-five participants were enrolled and completed the program, and all participants completed baseline and follow-up exploratory cognitive assessments. Adherence was defined as attending at least 16 sessions across the intervention period. Fifty-six participants (75%) met this adherence threshold. No adverse events occurred during the intervention period. These findings indicate that the long-duration, low-frequency DTT and EXG program is feasible to implement in a senior living community setting.

### 3.3 Cognitive outcome measures

All 75 participants who completed the program had baseline cognitive assessments, and 75 also completed follow-up testing approximately 12 months later. Thus, analyses of the MoCA, the CNS Vital Signs composite and its subtests, and subjective memory included paired baseline and follow-up data from 75 participants. Table 3 presents the baseline and post-intervention scores for participants, along with associated statistics and effect sizes (see also the Supplementary material for violin plots illustrating the distribution of outcomes at baseline and post-intervention). Significant improvements were observed in cognition as measured by MoCA (median change = 1.5, IQR = 2, *p* < 0.0001, *d* = 0.61) and CNS Vital Signs Composite score (median change = 3.5, IQR = 9, *p* < 0.0001, *d* = 0.45).

**Table 3:** Baseline and post-intervention scores.

Outcome measure * = significant outcome, *p* < 0.05	Baseline median (Range)	Post-intervention median (Range)	Wilcoxon signed rank test *p*-value	Effect size: Hodges–Lehmann median paired difference (95% CI)	Effect size: Cohen’s *d*
MoCA^*^	28 (17–30)	29 (18–30)	**<0.0001**	1.5 (1, 2)	0.61
CNSVS composite^*^	105.5 (64–120)	108 (74–126)	**<0.0001**	3.5 (2, 5)	0.45
Verbal memory	109.5 (54–130)	110 (73–130)	0.2	4 (−1.5, 8.5)	0.21
Visual memory	106 (42–130)	107 (83–130)	0.3	3 (−4, 9)	0.18
Motor speed (Finger tapping right)^*^	107 (51–125)	109 (47–125)	**0.01**	4 (1, 7.5)	0.30
Motor speed (Finger tapping left)	102 (31–125)	106 (40–141)	0.06	2.5 (0, 7)	0.25
Processing speed (Symbol digit coding)^*^	112.5 (58–140)	118 (77–140)	**0.05**	3.5 (0, 7)	0.31
Executive functioning (Stroop)	102 (67–115)	103 (72–114)	0.5	0.5 (−2, 4)	0.16
Executive functioning (Shifting attention)^*^	103 (52–125)	107 (54–125)	**0.02**	5 (1, 10)	0.34
Attention (Continuous performance)	105 (−251–106)	106 (14–106)	0.7	−17.5 (−58.5, 160.5)	0.06
Subjective memory	30 (0–111)	30 (0–93)	0.25	−3.5 (−11, 3.5)	−0.19

The bold and asterisk (*) represent statistically significant outcome measure, *p* < 0.05.

Further analyses determined the subscales of the CNS Vital Signs that contributed to the change in the Composite score. Motor speed, as measured by Finger Tapping Right (median change = 4, IQR = 11.25, *p =* 0.01, *d* = 0.30) and Symbol Digit Coding (median change = 3.5, IQR = 13, *p* = 0.05, *d* = 0.31), and executive function, as measured by Shifting Attention (median change = 5, IQR = 13.25, *p* = 0.02, *d* = 0.34), improved significantly. Improvement in Finger Tapping Left (median change = 2.5, IQR = 8, *p* = 0.06, *d* = 0.25) did not reach statistical significance. Verbal memory (*p* = 0.2), visual memory (*p* = 0.3), executive functioning as measured by the Stroop test (*p* = 0.5), and attention as measured by the Continuous Performance Test (*p* = 0.7) did not show significant changes. Subjective memory also did not change significantly (*p* = 0.3). No cognitive test scores declined significantly between baseline and post-intervention. Controlling for age, sex, and number of sessions completed did not change any of the above findings. As a sensitivity analysis, inferences for the three secondary exploratory outcomes remained unchanged when applying the Holm–Bonferroni correction across them.

Improvements in the cognitive outcomes were not significantly associated with the number of sessions completed (MoCA: Kruskal–Wallis *p* = 0.2; CNS Vital Signs: *p* = 0.4; subjective memory: *p* = 0.5). In the baseline-adjusted ordinal-dose model (post score ~ baseline score + sessions), we likewise found no evidence of a linear dose–response: the estimated coefficient was MOCA: *β* = 0.025 (95% CI, −0.12 to 0.09), *p* = 0.6; CNSVS Composite: *β* = 0.205 (95% CI, −0.03 to 0.63), *p* = 0.3; and subjective memory: *β* = −0.300 (95% CI, −0.92 to 0.59), *p* = 0.6.

## 4 Discussion

### 4.1 Feasibility

The primary aim of this single-arm feasibility study was to test the feasibility of a combined, low-frequency, long-duration DTT and EXG intervention in older adults living within a retirement community. Cognitive outcomes were considered secondary, exploratory endpoints in this single-arm feasibility study. The present single-arm feasibility study demonstrates that a long-term, facility-based program integrating DTT and EXG is feasible among older adults residing in a senior living community. High program adherence (75%) and absence of adverse events underscore the practicality and safety of delivering cognitive-motor interventions in community settings. This is consistent with prior findings indicating that older adults find exergaming interventions engaging, safe, and less physically strenuous than traditional exercise (Chao et al., 2014; Barry et al., 2016).

A possible contributor to the high adherence observed in this study was the integration of gamification components through the EXG, which introduced real-time feedback, performance scoring, and varied, goal-oriented tasks that may have enhanced motivation and adherence. Compared with DTT alone, EXG interventions provide stimuli that can make cognitively demanding physical activity feel more interactive and rewarding, potentially reducing monotony and perceived exertion. Prior research suggests that older adults often find exergaming more engaging and less strenuous than traditional exercise at similar intensities (Stojan and Voelcker-Rehage, 2019; Gouveia et al., 2020). Although enjoyment and acceptability were not formally assessed, they should be in future research to better understand the mechanisms supporting sustained participation in combined DTT and EXG interventions.

The implementation across 12 months further supports the feasibility of this intervention. Although the intervention spanned 12 months, the training dose was low-frequency, typically once weekly during program blocks. Thus, the feasibility demonstrated here pertains to a long-duration, low-dose model, which may differ from higher-frequency interventions reported elsewhere. Previous trials often focus on short-term (4–12 week) interventions (Law et al., 2014; Adcock et al., 2019), limiting understanding of longer-term sustainability and adherence in real-world settings. The intervention model used in the present single-arm feasibility study employed a structured approach that appears to promote sustained engagement, suggesting potential for integration into existing wellness programs within senior living communities.

### 4.2 Cognitive outcomes

It was hypothesized that participants would demonstrate improvements in objective cognitive measures as secondary exploratory outcomes. Although subjective memory performance did not significantly change over the 12-month period, overall objective cognitive performance did significantly improve, including global cognition and cognitive composite scores. The domain-specific cognitive functions that significantly contributed to this improvement were processing speed and executive functioning. The effect sizes for the outcomes with significant improvements ranged between 0.30 and 0.61. Consistent with other findings in DTT and EXG interventions, these results suggest that participation in such interventions improves processing speed and global cognitive functioning (Wollesen et al., 2020; Stanmore et al., 2017).

Improvements were observed primarily in processing speed and executive function domains. These findings are consistent with previous research suggesting that tasks require rapid responses, impulse control, and visual scanning (Tait et al., 2017; Herold et al., 2018). This may be significant given the role of executive functions in everyday life, and the gradual slowing of processing speed throughout the aging process (Denkinger et al., 2012; Law et al., 2020). The absence of significant change in memory measures may reflect the intervention’s lower memory demands. Although subjective memory ratings did not change significantly, it is possible that the participants’ high baseline education and cognitive status created a ceiling effect, masking perceived improvement.

Given that improvement was not seen across all cognitive measures, DTT and EXG may need to be more demanding and varied to optimize cognitive functioning. Further, in the present single-arm feasibility study, we did not observe a dose–response effect: improvements in cognitive performance in those participants who completed only 8 sessions were not significantly different from those who completed additional sessions (up to 24 sessions in total). The lack of a linear dose–response may indicate that cognitive gains plateau after a minimum level of engagement or that maintenance, rather than intensity, drives sustained benefit. It is also possible that the dose variability, in terms of number of sessions and period of time, could be regulated in future studies (e.g., one session per week across 8 consistent weeks), potentially amplifying the benefits seen from the program. Higher-frequency interventions may yield differential outcomes, which should be explored in future trials.

### 4.3 Study limitations

Despite encouraging results, several limitations should be acknowledged. The primary limitation of the single-arm feasibility study is the absence of a control group. The absence of a control group precludes causal inference regarding the observed cognitive improvements. This was a single-site program evaluation, with data collected by the primary interventionists, which may have introduced bias and limited external validity. The intervention was relatively low-dose and low-frequency over a longer duration, which may have constrained the magnitude of the observed outcomes. This single-arm feasibility study focused on DTT and EXG in older adults living in a community setting; as such, the generalizability of the results is limited to other populations and settings. The staff who facilitated the intervention also conducted the assessments, without blinding, thereby increasing the risk of bias. In addition to feasibility, the intervention’s acceptability was not formally measured.

Practice effects may partially explain improvements in repeated cognitive testing. Although the 12-month interval reduces short-term retest effects, the absence of a control group prevents differentiation between intervention-related change and potential test–retest effects. The observed median improvement of 1.5 points on the MoCA, while statistically significant, should be interpreted cautiously. Minimal clinically important differences for the MoCA remain debated, and future controlled trials are needed to determine their clinical relevance. Although the intervention lasted 12 months, session frequency was low compared with many exercise trials. Therefore, feasibility conclusions apply specifically to a long-duration, low-frequency model and should not be generalized to high-dose protocols.

Given the retirement community’s location in the United States (Gaithersburg, Maryland), there was minimal ethnic diversity (95% of participants identified as white). This was also a well-educated group, as all participants held a bachelor’s degree or higher. There was also no comorbidity or baseline physical activity data to better contextualize the results. An evaluation of prior physical activity, health status, prior experiences with DTT or EXG interventions, and digital literacy may influence cognitive outcomes and should be controlled for in future research. To better generalize the results of this single-arm feasibility study to all older adults, it would be beneficial to replicate it in a randomized controlled trial with a more diverse participant pool. Reliance on self-reported measures of subjective memory may have reduced sensitivity to detect meaningful change.

There are several barriers to implementing combined DTT and EXG programs in real-world settings. Similar to equipment-based fitness programs, this programmatic approach requires ample space and financial resources. In addition, available staff need to be trained to implement these programs and properly operate specific equipment, such as the SMARTFit, Dividat Senso, and LudoFit solutions. To the authors’ knowledge, this is the first single-arm feasibility study to combine multiple DTT and EXG interventions in a single program, although the concept of the “brain gym” has been previously published (Glatt et al., 2024). While this single-arm feasibility study supports the feasibility and preliminary effectiveness of such a program, more research is needed to further establish the efficacy of combined DTT and EXG programs across various settings.

## 5 Conclusion

This single-arm feasibility study demonstrated that a low-frequency, long-duration intervention integrating exergaming and dual-task training is feasible and associated with improvements in cognitive outcomes among older adults. The program provided a structured format for the simultaneous training of cognitive and physical functions, resulting in high adherence (75%). These findings support the potential of low-frequency, long-duration, facility-based multimodal interventions as a viable translational model for promoting cognitive health in aging populations. While findings are promising, results should be interpreted cautiously given the single-arm feasibility design and low intervention dose.

The integration of evidence-based dual-task and exergaming modalities within a structured “brain gym” program may represent an effective means of maintaining or improving cognitive functioning through sustained engagement in cognitively demanding physical activity. Future research employing randomized controlled designs should further evaluate the efficacy, optimal training dosage, and long-term retention of cognitive gains, as well as the potential secondary benefits for mobility and psychosocial wellbeing.

Beyond demonstrating feasibility and preliminary effectiveness, this single-arm feasibility study highlighted a model for embedding combined DTT and EXG programs into residential and community wellness settings. Such real-world translation represents a critical next step toward broad adoption of programs that support cognition in aging populations.

## Data Availability

The original contributions presented in the study are included in the article/Supplementary material, further inquiries can be directed to the corresponding author.
