# Supplementary material for: A facility-based “brain gym”: feasibility and preliminary effectiveness of a long-duration, low-frequency dual-task and exergaming intervention in older adults
Source: Front Psychol. 2026 Mar 26;17:1767634. doi: 10.3389/fpsyg.2026.1767634 (PMC13061681; doi:10.3389/fpsyg.2026.1767634)
Supplement: Supplementary file 1 [file Table_1.DOCX]

**Supplementary Materials 1 – The Memory Questionnaire (MEMQ) “Subjective Memory” Items**

The Memory Questionnaire (MEMQ) total score is calculated by summing responses across all items. Each item was rated on a Likert-type scale reflecting the frequency of subjective memory difficulties in everyday situations, with higher values indicating greater perceived difficulty. Item scores were coded so that higher total scores reflect more frequent or severe subjective memory concerns. The total score, therefore, represents a global index of subjective memory functioning. No subscales were calculated. Missing responses were not imputed; total scores were calculated only for participants with complete item responses. The MEMQ was used for descriptive and exploratory purposes and is not intended as a diagnostic instrument.

**
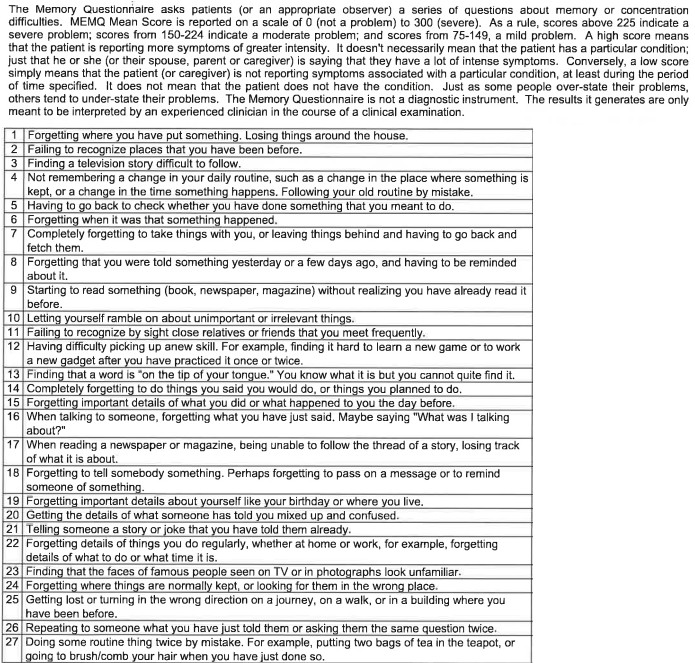
**
