# Supplementary material for: A facility-based “brain gym”: feasibility and preliminary effectiveness of a long-duration, low-frequency dual-task and exergaming intervention in older adults
Source: Front Psychol. 2026 Mar 26;17:1767634. doi: 10.3389/fpsyg.2026.1767634 (PMC13061681; doi:10.3389/fpsyg.2026.1767634)

**Supplementary Materials 2 -Distribution of outcomes at baseline and post intervention**

*** = significant outcome, p < .05**


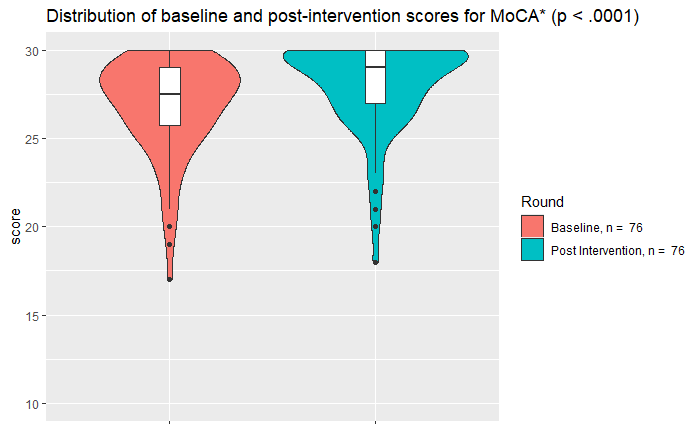


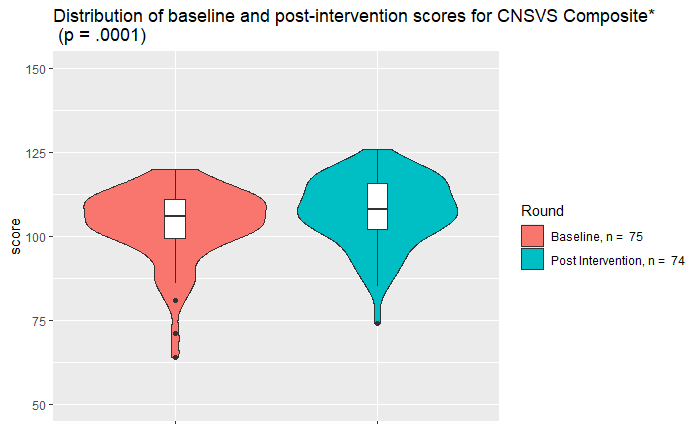


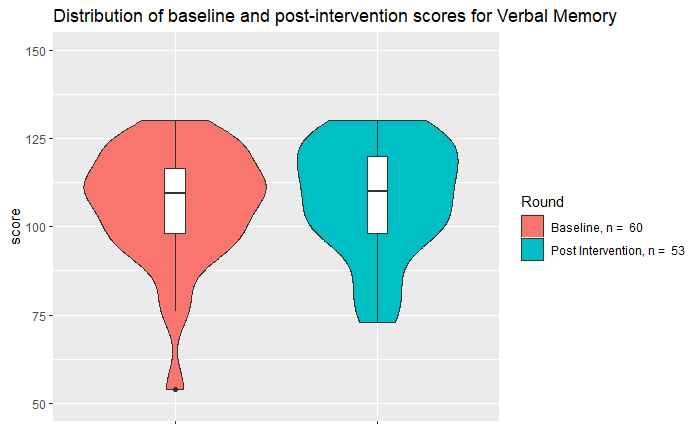


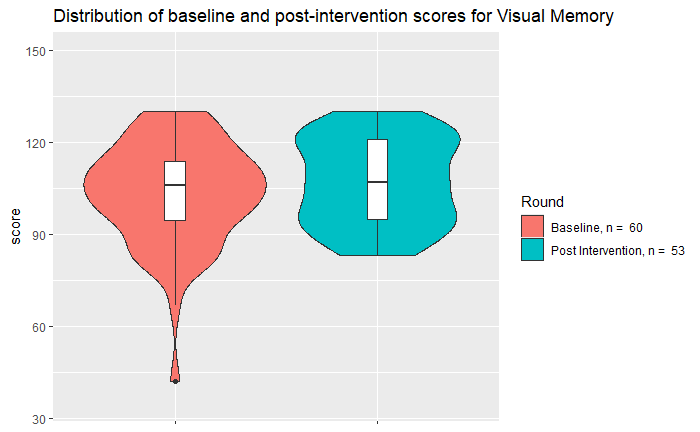


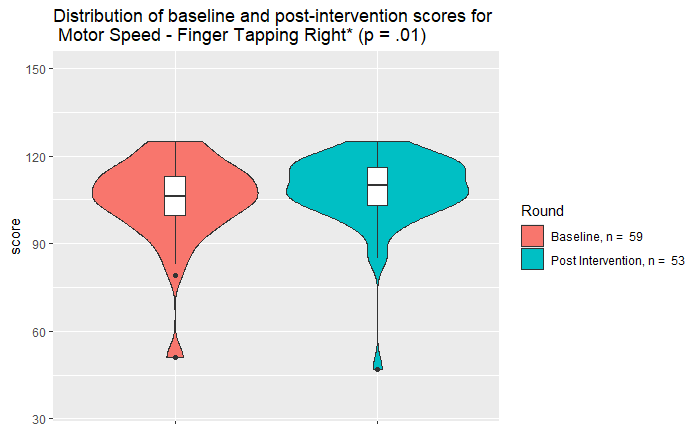


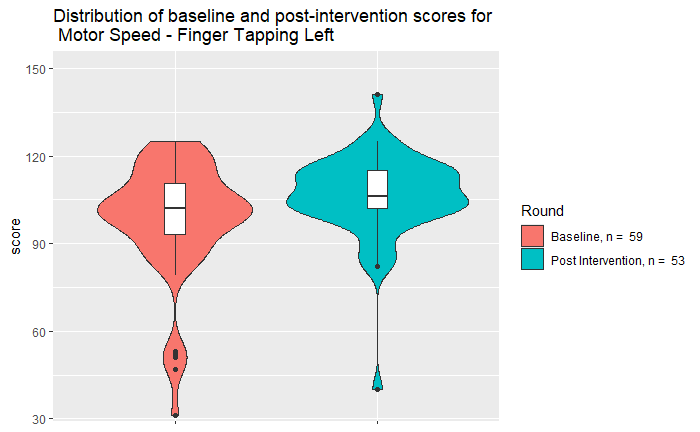


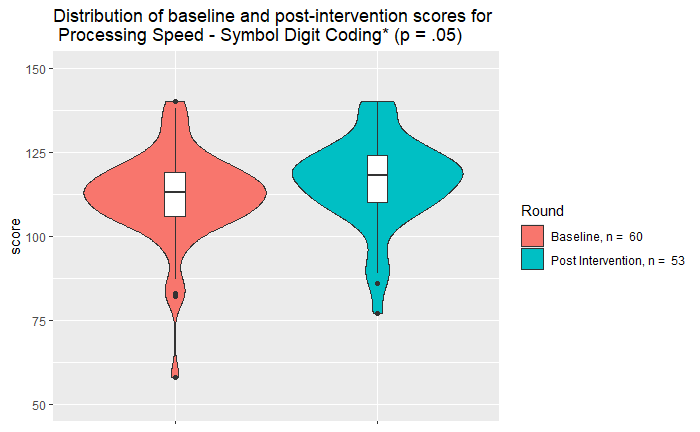


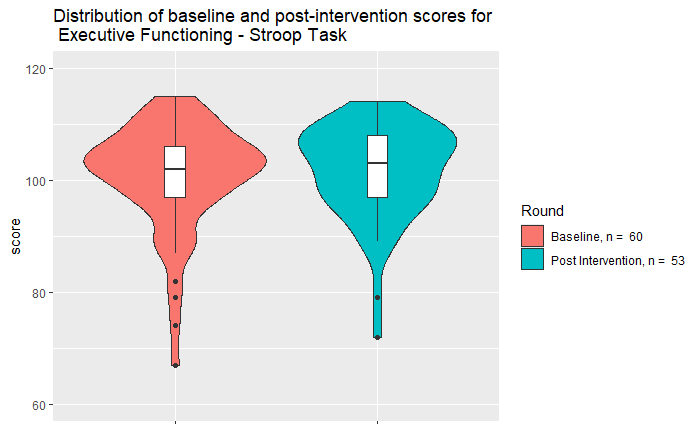


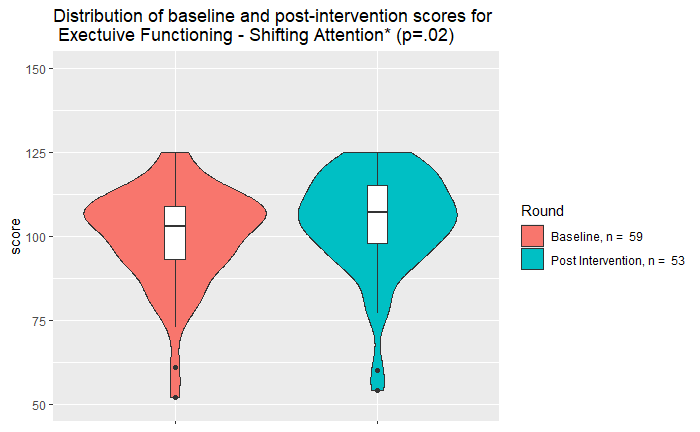


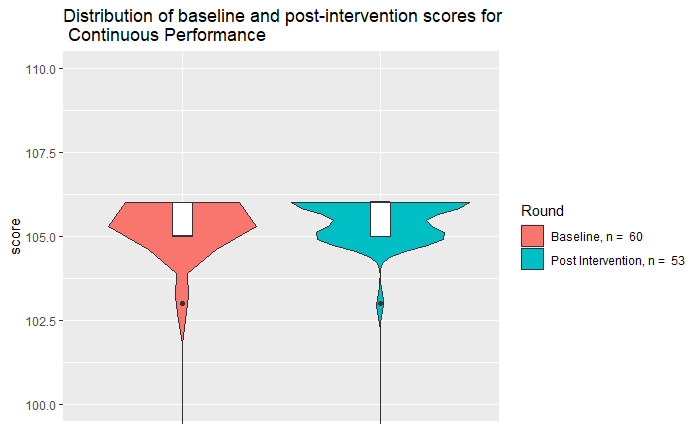


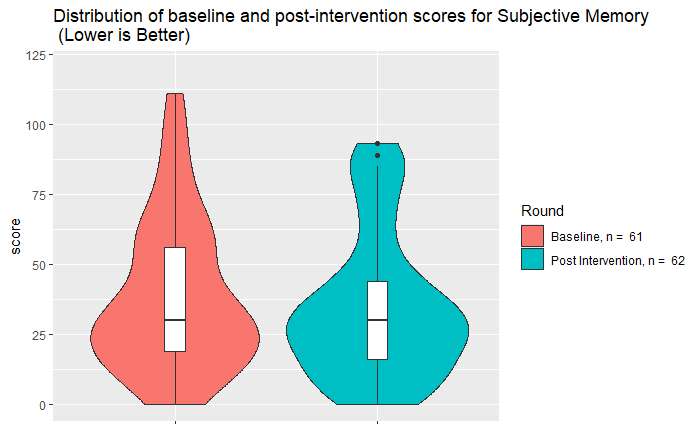

Supplement: Supplementary file 2 [file Table_2.DOCX]
