# Supplementary material for: A facility-based “brain gym”: feasibility and preliminary effectiveness of a long-duration, low-frequency dual-task and exergaming intervention in older adults
Source: Front Psychol. 2026 Mar 26;17:1767634. doi: 10.3389/fpsyg.2026.1767634 (PMC13061681; doi:10.3389/fpsyg.2026.1767634)
Supplement: Supplementary file 3 [file Table_3.DOCX]

**Supplementary Materials 3: Consensus on Exercise Reporting Template (CERT) Checklist**

| **CERT Item** | **Description** | **Reported in Manuscript** |
| --- | --- | --- |
| **1. Type of exercise equipment** | Detailed description of exercise equipment used | Methods – Intervention Section (SMARTFit system, Dividat Senso platform, LudoFit interface; manufacturer details provided) |
| **2. Qualifications of provider** | Instructor qualifications, training, and experience | Methods – Intervention Section (Certified Personal Trainer; oversight by licensed Occupational Therapist) |
| **3. Individual vs group delivery** | Whether exercise was delivered individually or in group format | Methods – Intervention Section (Group-based sessions in senior living community setting) |
| **4. Supervision** | Level and nature of supervision during sessions | Methods – Intervention Section (Continuous in-person supervision; staff-to-participant ratio approximately 1:6) |
| **5. Adherence measurement and strategies** | How adherence was monitored and strategies used to improve adherence | Methods – Feasibility Outcomes (Attendance recorded at each session; real-time feedback and instructor reinforcement) |
| **6. Setting** | Description of where exercise occurred | Methods – Intervention Section (Dedicated exercise room within senior living community with equipment in Item 1) |
| **7. Frequency of sessions** | How often sessions occurred | Methods – Intervention Section (Approximately one 60-minute session per week during each 8-week block) |
| **8. Duration of sessions** | Length of each session | Methods – Intervention Section (60 minutes per session) |
| **9. Total program duration** | Overall duration of the intervention | Methods – Intervention Section (12 months consisting of two structured 8-week blocks with maintenance periods) |
| **10. Exercise intensity** | Description of physical and cognitive intensity | Methods – Intervention Section (Moderate intensity; RPE 11–14; cognitive load progressed when ≥80% task accuracy achieved) |
| **11. Progression rules** | Criteria used to progress exercises | Methods – Intervention Section (Progression when participants achieved ≥80% task accuracy with stable balance) |
| **12. Tailoring / individualization** | How exercises were adapted for individuals | Methods – Intervention Section (Modification of stepping speed, stimulus complexity, dual-task demands, and balance challenge based on performance and safety) |
| **13. Starting level decision** | How initial difficulty was determined | Methods – Intervention Section (Initial task difficulty selected based on baseline cognitive and mobility performance) |
| **14. Fidelity monitoring** | Procedures to ensure consistent delivery | Methods – Intervention Section (Standardized session templates; oversight by occupational therapist; structured format) |
| **15. Adverse events** | Monitoring and reporting of adverse events | Results – Feasibility Outcomes (No serious adverse events) |
| **16. Non-exercise components / home program** | Description of additional components | Methods – Intervention Section (Intervention fully supervised and facility-based) |
